# AbdomenNet: deep neural network for abdominal organ segmentation in epidemiologic imaging studies

**DOI:** 10.1186/s12880-022-00893-4

**Published:** 2022-09-17

**Authors:** Anne-Marie Rickmann, Jyotirmay Senapati, Oksana Kovalenko, Annette Peters, Fabian Bamberg, Christian Wachinger

**Affiliations:** 1 Lab for Artificial Intelligence in Medical Imaging, Department of Medicine, Ludwig Maximilians University Munich, Munich, Germany; 2 Lab for Artificial Intelligence in Medical Imaging, Institute of Diagnostic and Interventional Radiology, Technical University of Munich, Munich, Germany; 3 Institute of Epidemiology, Helmholtz Zentrum Munich, Munich, Germany; 4 Department of Diagnostic and Interventional Radiology, Medical Center-University of Freiburg, Faculty of Medicine, University Freiburg, Freiburg, Germany

**Keywords:** Deep learning, Segmentation, Abdominal MRI, Dixon MRI

## Abstract

**Background:**

Whole-body imaging has recently been added to large-scale epidemiological studies providing novel opportunities for investigating abdominal organs. However, the segmentation of these organs is required beforehand, which is time consuming, particularly on such a large scale.

**Methods:**

We introduce AbdomentNet, a deep neural network for the automated segmentation of abdominal organs on two-point Dixon MRI scans. A pre-processing pipeline enables to process MRI scans from different imaging studies, namely the German National Cohort, UK Biobank, and Kohorte im Raum Augsburg. We chose a total of 61 MRI scans across the three studies for training an ensemble of segmentation networks, which segment eight abdominal organs. Our network presents a novel combination of octave convolutions and squeeze and excitation layers, as well as training with stochastic weight averaging.

**Results:**

Our experiments demonstrate that it is beneficial to combine data from different imaging studies to train deep neural networks in contrast to training separate networks. Combining the water and opposed-phase contrasts of the Dixon sequence as input channels, yields the highest segmentation accuracy, compared to single contrast inputs. The mean Dice similarity coefficient is above 0.9 for larger organs liver, spleen, and kidneys, and 0.71 and 0.74 for gallbladder and pancreas, respectively.

**Conclusions:**

Our fully automated pipeline provides high-quality segmentations of abdominal organs across population studies. In contrast, a network that is only trained on a single dataset does not generalize well to other datasets.

## Background

Whole-body magnetic resonance imaging (MRI) is increasingly used in large-scale imaging studies, for instance, in ongoing population-based studies like the German National Cohort (GNC) [1, 2] and the UK Biobank Imaging (UKB) [3], as well as earlier studies like the Study of Health in Pomerania (SHIP) [4] or the Kohorte im Raum Augsburg (KORA) [5, 6]. Whole-body MRI provides rich anatomical information across the body, which in combination with physiological, biochemical, genetic, and demographic data on a large scale, provides unique opportunities for advancing our understanding of the human body. The data may provide novel insights into disease mechanisms or facilitate the identification of imaging measures as targets for prevention. Moreover, it will allow for mapping out normal anatomical variations and for detecting asymptomatic pathology before disease diagnosis. A requirement for many of such analyses is, however, the automated extraction of quantitative measures. While sophisticated pipelines have been developed for this task in brain MRI [7, 8], they are still widely lacking in abdominal MRI. Yet, with the growing availability of whole-body datasets, such tools will be needed.

Given that several whole-body datasets are emerging, a key concern is to have a processing pipeline that can not only be applied to data from a single dataset but from multiple datasets. Such a common segmentation model will support joint analyses across datasets and can therefore help to grow the sample size or to replicate findings. Previous studies have demonstrated that extracted image features can be subject to dataset bias, which impedes joint analyses across datasets [9]. Individual segmentation models that are only trained on a single dataset are more likely to introduce bias than a model that is trained across datasets. Hence, we are interested in a segmentation pipeline that operates across datasets. To address these challenges, we build upon recent advances in deep neural networks and present a segmentation pipeline that automatically segments eight abdominal organs across datasets. In a pre-processing step, we merge several MRI scans from the two-point Dixon sequence to cover the abdominal area. Moreover, inhomogeneity correction, stitching, resampling, and an optional intensity standardization are applied to the scans. The pipeline automatically processes all four contrasts from the Dixon sequence and yields a standardized output across datasets, which will then be the input to the segmentation network.

At the core of the pipeline is the AbdomenNet, a deep neural network that is inspired from earlier fully convolutional neural networks with skip connections like the U-Net [10] and QuickNAT [11]. The inputs to the network are multiple contrasts from the Dixon sequence. Technical innovations of AbdomenNet are the integration of octave convolutions [12] in the network to reduce the spatial redundancy in convolutional neural networks while increasing the receptive field. Furthermore, octave convolutions are combined with squeeze and excite blocks [13, 14] for feature recalibration, which helps the network to focus on important features, while only marginally increasing network complexity. Finally, AbdomenNet uses stochastic weight averaging (SWA) [15] in the optimization, which can provide solutions that improve generalization.

As mentioned before, we are not only interested in processing data from a single cohort but from multiple cohorts to support joint analyses. Specifically, we work with data from GNC, UKB, and KORA. Whole-body scans from all three studies are pre-processed and used to train a joint segmentation network.

### Related work

Automated methods for the segmentation of multiple abdominal organs have mainly been proposed for CT scans. The segmentation of CT scans, in comparison to MRI scans, is easier as the intensity values are standardized in Hounsfield units and the images generally have higher image quality and resolution. Traditional methods like thresholding [16], region growing [17] or atlas based segmentation methods [18] have been successfully used in the past, but with the breakthrough in deep learning, the focus has shifted to using convolutional networks for multi-organ segmentation. Recent methods for CT segmentation proposed a two-stage hierarchal pipeline using 3D U-Net [19], the segmentation of 2D slices with fusion and organ-attention [20], the inclusion of organ size as prior [21], and the application of V-net [22]. Approaches for the segmentation of multiple organs in MRI have utilized the 2D U-Net [23] and 2D fully convolutional networks [24]. In our prior work on abdominal segmentation, we have proposed to recalibrate feature maps on CT scans [14, 25] and to estimate the segmentation uncertainty for liver segmentation on MRI [26].

As self-reported results in publications are typically difficult to compare because of variations in the experimental setup and data, the results from challenges provide a good overview of the state of the field. Multi-organ segmentation has been evaluated in three challenges over the last years: the VISCERAL challenge in 2014 [27], the Multi-Atlas Labeling Beyond the Cranial Vault (MAL) challenge in 2015, and the Combined (CT-MR) Healthy Abdominal Organ Segmentation (CHAOS) challenge in 2019 [28]. Most relevant for application is task 5 in CHAOS, which was on the segmentation of the liver, kidneys, and spleen in MRI. The winning method used nnU-Net [29], which is an automated and robust training scheme for U-Net. The conclusion from the CHAOS challenge was that networks have reached inter-expert variability in Dice score for liver CT segmentation, but multi-organ segmentation is more challenging for MRI.

## Methods

### Datasets

**Fig. 1:**
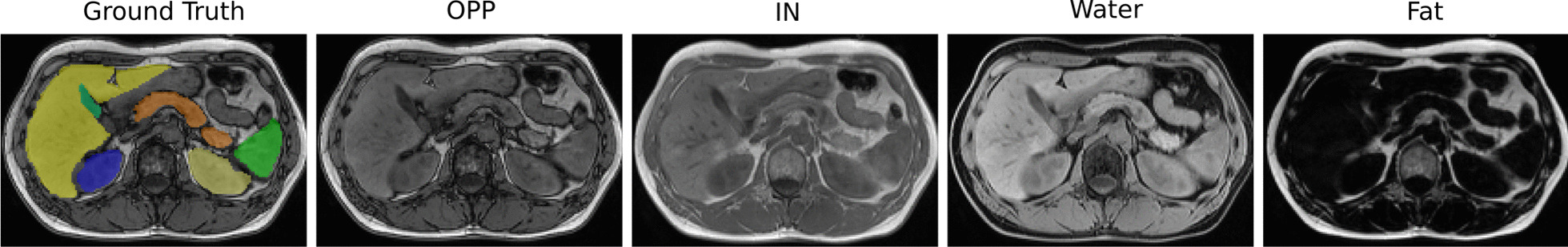
Dixon contrasts: Ground truth segmentation overlayed on the OPP contrast and all four Dixon contrasts of a scan from the GNC study. OPP and Water contrasts provide the clearest depiction of the organs

**Fig. 2:**
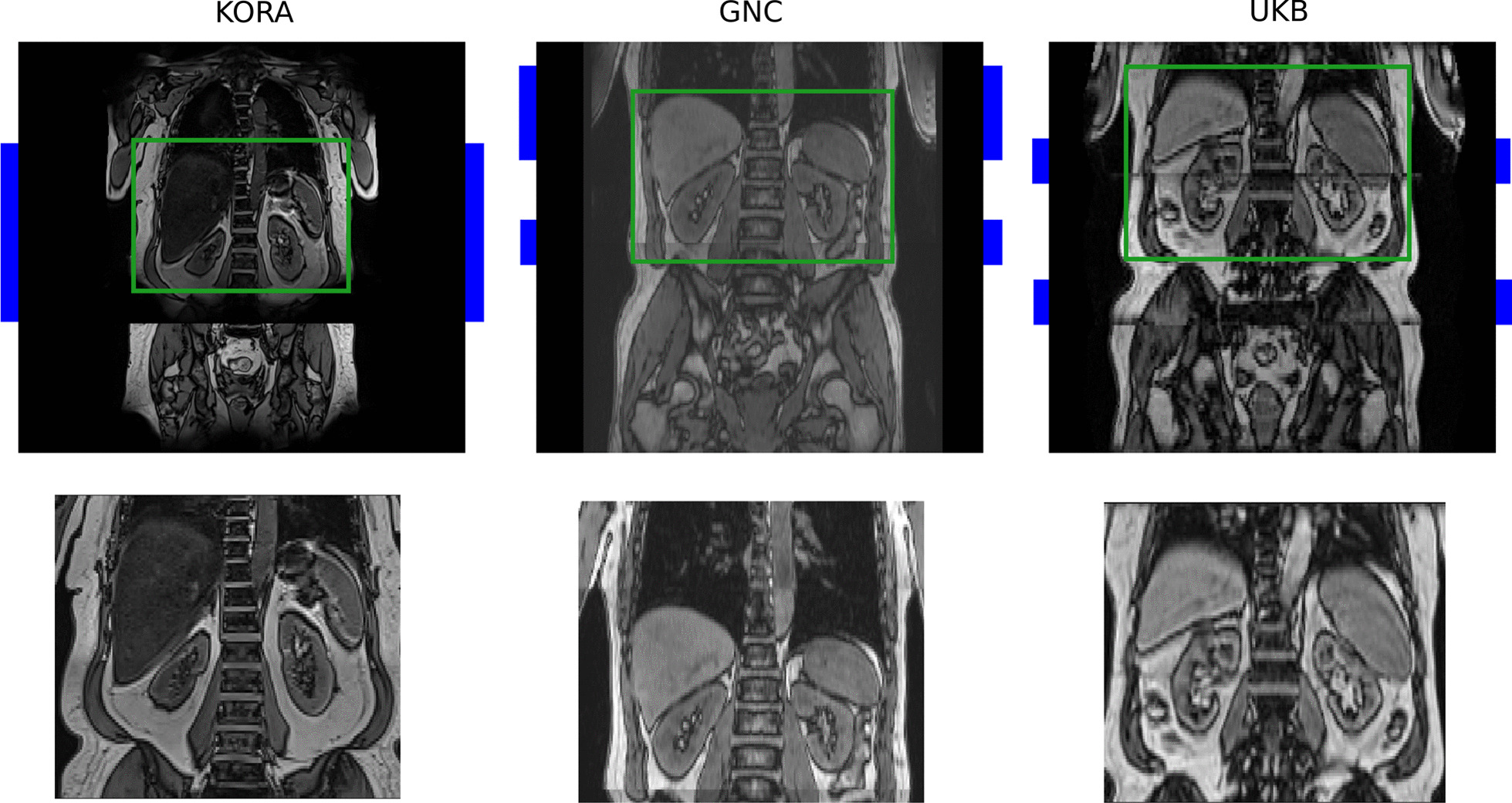
Illustration of MRI scans from different datasets. Top row: original OPP scans, with overlapping regions indicated by blue bars and regions of interest indicated by green boxes. Bottom row: standardized scans as output of the pre-processing pipeline

We work with data from three cohort studies that include whole-body MR imaging: the German National Cohort (GNC) [1], the UK Biobank Imaging (UKB) [3], and the Kohorte im Raum Augsburg (KORA) [5]. GNC and UKB are still in the process of acquiring data with the goal of scanning 30,000 and 100,000 subjects, respectively. KORA is already completed and includes 400 subjects. All three studies acquired abdominal images with a T1-weighted 3D volumetric interpolated breath-hold examination (VIBE) two-point Dixon sequence with participants in supine position. The Dixon sequence results in four sets of images: water only, fat only, in-phase (IN), and opposed-phase (OPP), illustrated in Fig. 1. OPP scans from all three datasets are illustrated in Fig. 2. In our experiments, we investigate the segmentation performance on all four contrasts. The types of scanners differ between datasets with a 3T Siemens Skyra for GNC and KORA, and a 1.5T Siemens Aera for UKB. Further, the acquisition details, e.g., echo time, repetition time, and field of view (FOV), vary between the datasets. The FOV is the smallest for UKB, which requires merging more MRI stages at different table positions to cover the region that contains all organs of interest. Our automated pre-processing is described in the next section.

**Fig. 3:**
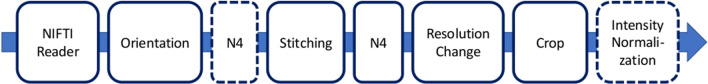
Our proposed pre-processing pipeline reads abdominal MRI files from different stages, changes the read-orientation to RAS, stitches the scans, performs bias field correction, resamples to a standard resolution, and crops out the ROI covering abdominal organs of interest. Optional steps are depicted with dashed lines

**Fig. 4:**
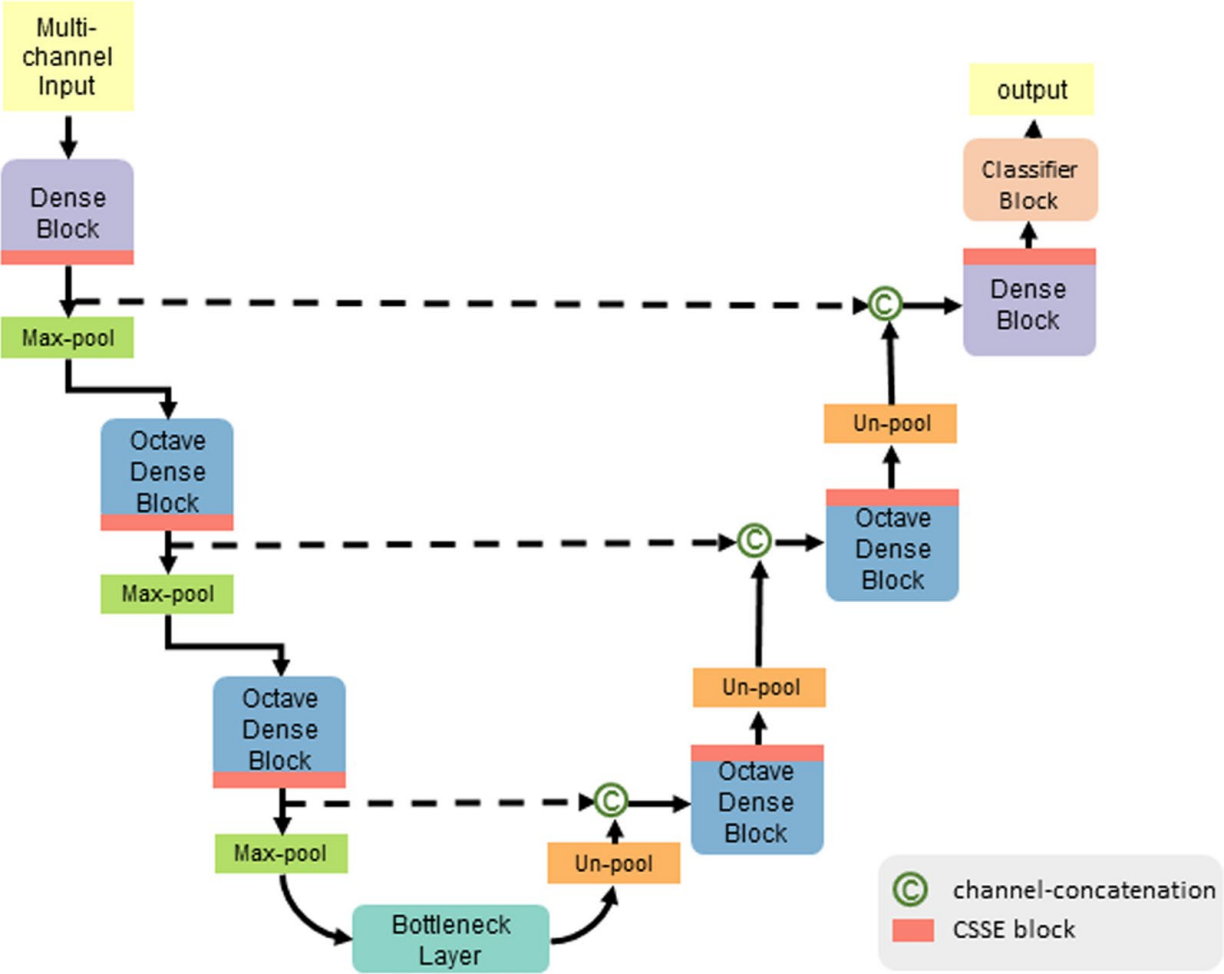
The network architecture, based on QuickNAT [11], with additional CSSE blocks in each layer and Octave Dense Blocks

**Fig. 5:**
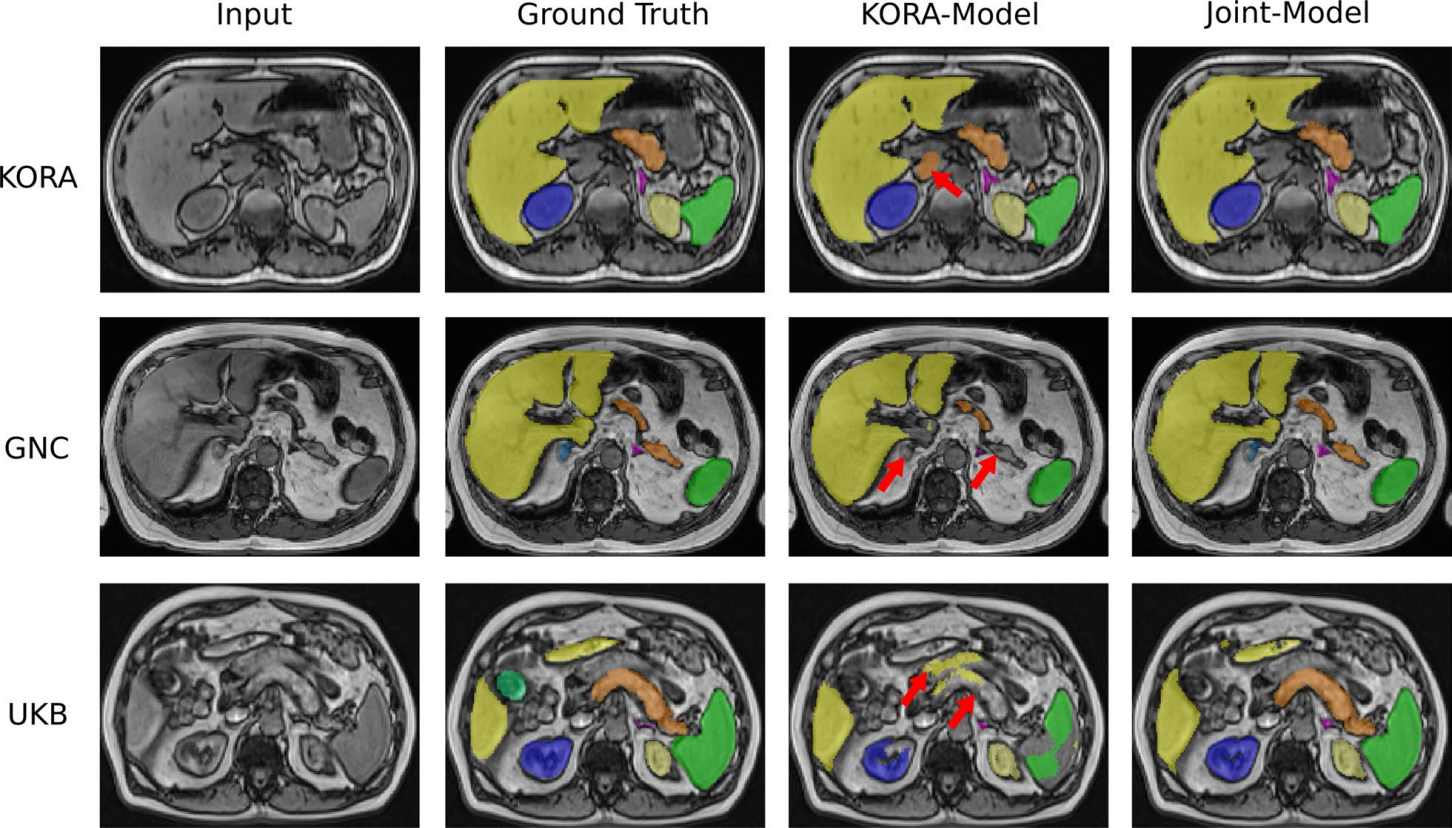
Segmentation results of axial AbdomenNet trained on axial view on KORA and Joint dataset, with testing results on KORA, GNC, and UKB (rows). Red arrows point to false segmentations and missed segmentations

For training and evaluation, 61 scans have been manually segmented by a trained anatomist. These scans have been selected based on demographics (age, sex) and BMI to have a heterogeneous sample that captures the variability in the data. Table 1 reports the statistics of the selected scans for manual annotation.

**Table 1:** Descriptive statistics of the selected subjects for the three datasets

	KORA	GNC	UKB
$\#$ Subjects	18	17	25
Sex (M/F)	11/8	8/9	12/13
Age	$56.9 \pm 9.4 \ (40.0 / 72.0)$	$52.4 \pm 13.7 \ (22.3 / 69.6)$	$66.0\pm 6.6$ (49.3/76.4)
Height	$171.9 \pm 11.3 \ (151.2 / 186.5)$	$171.1 \pm 9.3 \ (157.0 / 185.9)$	$167.0 \pm 8.6 \ (151.0 / 180.0)$
Weight	$83.4 \pm 13.4 \ (50.3 / 108.9)$	$83.5\pm 16.9 \ (57.2 / 127.2)$	$78.3 \pm 17.3 \ (55.0 / 113.0)$
BMI	$28.3 \pm 4.5 \ (22.0 / 35.9)$	$28.6 \pm 5.9 \ (21.7 / 39.6)$	$27.9 \pm 4.9 \ (21.3 / 40.9)$

For continuous values, we report mean, standard deviation, and min/max

### Pre-processing pipeline

The three datasets vary in image resolution, acquisition protocols, and the number of scanning stages, which is shown in the top row of Fig. 2. To facilitate the training of a neural network, we developed a fully automated, pre-processing pipeline that standardizes whole-body scans from the three datasets, where the different steps are shown in Fig. 3. The inputs for one subject are multiple MRI volumes from different stations. The output is a standardized image that is cropped to a region that contains the organs of interest. In detail, the images are loaded and all scans are transformed to a consistent scanning direction. Next, an optional N4 bias correction [30] is computed on the in-phase scans and the bias field is applied on all the individual scans, as done in [31]. Using the information about the table position, the scans are then stitched together. In the overlapping region, intensities from both scans are blended using a sigmoid function, given less weight to pixels close to the boundary as they are rather subject to artifacts. On the combined volume, N4 bias field correction is computed on the in-phase scans and the bias field is applied to all scans and the resolution is changed to $2 \times 2 \times 3$ mm$^3$.

The Dixon sequence covers a much larger region of the body, than the abdominal organs that we are segmenting. Hence, to facilitate the segmentation with the neural network, we define a cropping region across all datasets. To this end, we looked at the organ positions of the smallest/tallest and skinniest/fattest subjects and set the cropping region so that all organs are included. Once manually defined, the same cropping region is applied to all scans across the datasets. Optionally, we apply an intensity normalization as a final step, where we have implemented histogram matching and a linear intensity transform. For histogram matching, we randomly chose a scan from each dataset as reference when training single dataset models. For the joint model we chose a scan from the NAKO dataset, as NAKO scans have the best overall image quality. However, we have not noticed an improvement for either of the intensity normalization methods in our experiments so that we have not considered it further in our experiments. Yet, for other applications, such an intensity normalization could be beneficial.

### Segmentation model

For the segmentation of the pre-processed images, we introduce the AbdomenNet, which has an encoder/decoder architecture similar to QuickNAT [11] and U-Net [10]. Figure 4 illustrates the architecture of AbdomenNet, which has 3 encoders, 3 decoders separated by a bottleneck layer, and followed by a classifier block with a soft-max layer. The architecture includes skip-connections between all encoder and decoder blocks of the same spatial resolution. In the decoder stages, we use un-pooling for up-sampling the feature maps [32], which ensures the appropriate spatial mappings of activation maps. Un-pooling is particularly helpful for the segmentation of small structures, like abdominal glands. AbdomenNet can take multi-channel image input to process different contrasts from the two-point Dixon sequence.

In AbdomenNet, we use dense connections [33], which help representation learning by promoting feature re-usability and by providing a path for gradients to flow for better trainability. Each dense block consists of 3 convolutional layers, where a concatenation layer merges the outputs of the first and second convolutional layers. Before each convolutional layer, we use batch-normalization layers and parametric rectifier linear unit (PReLU) layers [34]. We have also experimented with group- and instance-normalization [35, 36], but have not noted an improvement in our experiments. We further use dropout with a dropout rate of 0.2 after each dense block.

#### Octave convolutions with squeeze-and-excite

AbdomenNet combines Octave Convolutions and Squeeze-and-Excite blocks, which we have not yet seen so far. The main idea of octave convolutions (OctConvs) [12] is to factorize feature maps along the channel dimension by their frequencies. Thus, lower frequency components can be stored with less resolution, which reduces memory and computational cost. Octave Convolutions can increase segmentation performance, by having a wider context, while reducing memory consumption. In our network, we replaced standard convolutions with Octave Convolutions in all layers of the network, except for the first encoder and last decoder block, as shown in Fig. 4.

We have further added squeeze-and-excite (SE) blocks [13] for the recalibration of feature maps. In particular, we use spatial and channel SE blocks (CSSE), which have been introduced for image segmentation [14]. In contrast to the original SE blocks, they do not only perform a channel recalibration but also a spatial recalibration. This is helpful for segmentation, where the objective is to find the spatial location of organs.

#### Loss function

The loss function of AbdomenNet consists of a weighted logistic loss and multi-class Dice loss. As we perform a slice-wise 2D segmentation not all organs are present in all slices. The Dice score for a true negative prediction is defined as zero, which would be penalized, using the standard Dice loss. We add 1 to the numerator and denominator of the Dice score to handle these cases. Given the estimated probability $p_l({\mathbf {x}})$ at pixel ${\mathbf {x}}$ to belong to the class *l* and the ground truth probability $g_l({\mathbf {x}})$, the loss function is

1 $$\begin{aligned} {\mathcal {L}} = \underbrace{ -\sum _{{\mathbf {x}}} \omega ({\mathbf {x}}) g_l({\mathbf {x}}) \log (p_{l}({\mathbf {x}}))}_{\mathrm {Logistic Loss}} - \underbrace{\frac{2 \sum _{{\mathbf {x}}} p_{l}({\mathbf {x}}) g_{l}({\mathbf {x}})+1}{\sum _{{\mathbf {x}}} p_{l}({\mathbf {x}}) + \sum _{{\mathbf {x}}} g_{l}({\mathbf {x}}) +1}}_{\mathrm {Dice Loss}}. \end{aligned}$$

We use weight factors $\omega ({\mathbf {x}})$, as introduced in [11], to give higher weight to small organs and organ boundaries.

#### Multi-view aggregation

The AbdomenNet consists of three 2D networks that operate on axial, sagittal, and coronal views, respectively. The results of these three networks are then combined in a view-aggregation step to retain more 3D information. An alternative to view-aggregation of 2D networks would be the application of a 3D network. However, due to the large field-of-view of abdominal scans and limited GPU memory, working with 2D networks allows for more architectural flexibility, e.g., dense blocks. In AbdomenNet, we give each anatomical view the same weight and average the probability scores of the individual networks. The probability score for a particular structure reflects the certainty of the network in the prediction, which depends on how well the structure is represented in the corresponding view. Aggregating all the votes for a voxel provides a regularization effect for the label prediction and thus reduces spurious predictions.

#### Model learning

We use stochastic weight averaging (SWA) [15] for training the AbdomenNet. SWA performs an equal average of the weights traversed by Stochastic Gradient Descent (SGD) with a modified learning rate schedule. SWA solutions end up in the center of a wide flat region of loss, while SGD tends to converge to the boundary of the low-loss region, making it susceptible to the shift between train and test error surfaces. Hence, SWA can improve generalization in deep learning over SGD.

## Results

### Experimental setup

The AbdomenNet models have 64 convolutional filters with kernel size 5 in each Dense Block. The $\alpha$ parameters for Octave Convolutions, which define how to split up the channels into high- and low-frequency components are set to 0.5. We implemented the network using PyTorch version 1.7.0. All AbdomenNet models were trained using SGD optimizer and SWA, with an SWA learning rate of 0.05. The batch size was set to 10 and we used PyTorch’s default weight initialization for initializing all learnable weights. As the models usually converged at around 70–80 epochs, we trained all models for 100 epochs and saved the model with the highest Dice Score on the validation set. For experiments validating the architecture and combination of Dixon contrasts, we split the annotated data randomly into training, validation, and testing set as follows: UKB (21/2/2), KORA(15/1/2), GNC(14/1/2). Each model was trained 3 times with different random seed values to enforce different weight initializations. We report the average performance of those 3 models to have a reliable estimate of the performance. To evaluate the effect of training models on single datasets and combinations of the datasets, we performed 10 fold cross-validation. The source code for the pre-processing pipeline and the segmentation network is available.^1^

### Evaluation across datasets

In the first experiment, we train separate segmentation models for each of three datasets, combinations of 2 datasets and a joint model. All models were trained on coronal, axial and sagittal views and the predictions of the three models were aggregated. To obtain reliable results, We performaed 10-fold cross-validation. The purpose of this experiment is to evaluate the generalizability of models across datasets. In Table 2, we list the training datasets vertically, and the test datasets horizontally and report average Dice coefficients and average symmetric surface distances. For instance, training on KORA and testing on KORA yields an average Dice score of 0.697, while training KORA and testing GNC yields 0.644. For the joint model, we merge the training and validation sets across the three datasets. The results for using a single dataset for training show the best results on the diagonal, which means that the same dataset is used for training and testing. We further observe that the transfer of models across datasets yields a strong decrease in accuracy. Particularly, when training on either KORA or GNC yields poor results on UKB. Next, we trained models on a combination of 2 datasets, and evaluated on all 3 datasets. We can observe, that the generalization ability of the networks increases over single-data models. For instance, The model trained on KORA+GNC yields better results on UKB, than models trained on KORA or GNC separately. We observe that a model trained on KORA+GNC improves performance on GNC and KORA, compared to the single dataset models.

**Table 2:** Mean Dice Scores and average symmetric surface distance (ASSD) in mm over all classes for multi-view AbdomenNet models trained on single datasets versus combinations of two and of all three datasets

	Dice			ASSD		
	KORA	GNC	UKB	KORA	GNC	UKB
KORA	0.697 ± 0.109	0.644 ± 0.075	0.385 ± 0.095	2.164 ± 0.758	3.287 ± 1.940	7.440 ± 3.519
GNC	0.637 ± 0.120	0.682 ± 0.089	0.311 ± 0.130	2.923 ± 1.018	2.899 ± 2.121	8.768 ± 7.267
UKB	0.433 ± 0.128	0.401 ± 0.143	0.610 ± 0.114	6.273 ± 3.074	7.831 ± 3.511	3.518 ± 3.634
KORA + GNC	0.728 ± 0.106	0.710 ± 0.073	0.498 ± 0.111	**1.658** ± 0.709	2.382 ± 1.762	3.780 ± 1.468
KORA + UKB	0.712 ± 0.112	0.683 ± 0.080	0.625 ± 0.119	2.036 ± 0.932	2.674 ± 1.726	3.238 ± 3.830
UKB + GNC	0.689 ± 0.106	0.697 ± 0.088	0.622 ± 0.116	1.757 ± 0.469	2.245 ± 1.533	5.074 ± 3.630
Joint Model	**0.731** ± 0.109	**0.716** ± 0.077	**0.637** ± 0.119	1.780 ± 0.818	**2.190** ± 1.559	**3.021** ± 3.666

Bold numbers indicate highest Dice scores and lowest ASSD scores

Finally, the joint model leads to the best performance across all three datasets. Noteworthy, the accuracy of the joint model is even higher than for the specialized models, e.g., training and testing on KORA. We show qualitative segmentation results comparing the model trained on KORA with the joint model in Fig. 5.

### Evaluation of Dixon contrasts

The Dixon sequence yields four different imaging contrasts: fat, water, in-phase (IN), and opposed-phase (OPP). We trained AbdomenNet models on the axial view of the joint dataset for each of the four contrasts and present the segmentation accuracy for all organs in Table 3. As OPP and Water contrasts show the most promising results, we further evaluate the combination of OPP and Water, by concatenating the images in the channel dimension. Considering the results per organ, the combination of OPP and water can improve over using the contrasts separately in several cases. However, there are also organs, where the combination yields results in between the individual contrasts. Nevertheless, the combination of OPP and water yields the highest mean accuracy. Hence, we use the combination of OPP and Water as input in the remaining experiments.

**Table 3:** Dice Scores and average symmetric surface distance (ASSD) in mm over all organs for axial AbdomenNet trained on different Dixon contrasts together with the mean scores across all organs

	Liver	Spleen	r.Kidney	l.Kidney	r.Adrenal	l.Adrenal	Pancreas	Gallbladder	Mean
*Dice*									
OPP	0.936 ± 0.035	**0.913** ± 0.036	0.845 ± 0.027	0.884 ± 0.035	**0.519** ± 0.084	0.525 ± 0.174	0.619 ± 0.187	**0.667** ± 0.229	0.739 ± 0.076
IN	0.936 ± 0.014	0.900 ± 0.036	0.901 ± 0.024	0.899 ± 0.040	0.398 ± 0.234	0.376 ± 0.192	0.538 ± 0.171	0.579 ± 0.140	0.691 ± 0.084
Fat	0.926 ± 0.020	0.849 ± 0.108	0.906 ± 0.030	0.906 ± 0.032	0.407 ± 0.235	0.409 ± 0.216	0.628 ± 0.146	0.514 ± 0.125	0.693 ± 0.081
Water	0.938 ± 0.019	0.906 ± 0.031	0.848 ± 0.048	0.846 ± 0.090	0.441 ± 0.246	**0.534** ± 0.160	**0.711** ± 0.095	0.628 ± 0.293	0.732 ± 0.082
OPP+W	**0.946** ± 0.020	0.905 ± 0.038	**0.918** ± 0.019	**0.908** ± 0.038	0.474 ± 0.187	0.466 ± 0.131	0.680 ± 0.135	0.627 ± 0.164	**0.741** ± 0.083
*Average symmetric surface distance (mm)*									
OPP	1.534 ± 1.269	1.346 ± 0.926	**0.877** ± 0.301	**0.822** ± 0.367	**1.480** ± 0.485	2.034 ± 1.446	**3.217** ± 1.571	**2.286 ± 1.784**	**1.700** ± 0.730
IN	1.698 ± 0.665	**1.275** ± 0.568	1.108 ± 0.273	1.077 ± 0.464	4.219 ± 6.048	4.073 ± 3.701	4.314 ± 1.497	3.275 ± 1.353	2.630 ± 1.272
Fat	2.127 ± 0.942	2.139 ± 1.294	1.023 ± 0.328	0.969 ± 0.406	2.280 ± 1.620	3.323 ± 3.092	3.558 ± 1.278	3.679 ± 1.062	2.387 ± 0.837
Water	5.246 ± 3.751	7.024 ± 5.970	8.580 ± 4.235	10.513 ± 7.873	4.149 ± 4.130	**1.840** ± 1.235	5.026 ± 3.748	7.629 ± 6.741	6.251 ± 0.949
OPP+W	**1.496** ± 0.849	1.380 ± 0.779	1.020 ± 0.325	0.959 ± 0.300	1.550 ± 0.658	2.887 ± 1.081	3.359 ± 1.469	3.035 ± 1.897	1.964 ± 0.576

Bold numbers indicate highest Dice scores and lowest ASSD scores

### Evaluation of network architecture

Table 4 reports the final performance of AbdomenNet with view aggregation across axial, coronal, and sagittal views. Note that in earlier experiments, we have only used models trained on axial views. To evaluate the impact of Octave Convolutions and CSSE blocks, we perform an architecture ablation study. We first train a model without Octave Convolutions and without CSSE, which results in the QuickNAT model [11]. Next, we add Octave Convolutions to QuickNat. The final addition of CSSE blocks results in AbdomenNet. We observe, that AbdomenNet outperforms the two other models on 5 out of 8 organs. Octave Convolutions and CSSE blocks seem to have the highest impact on gallbladder segmentation, where we observe an increase of 0.116 on the Dice score. We can observe a slight drop in performance on some organs (kidneys, adrenal glands) when adding Octave Convolutions, which could be due to the additional downsampling step. Adding additional CSSE blocks can help in these cases. To validate the impact of training with SWA, we trained an AbdomenNet model with SGD instead of SWA and observed decreased performance with an average Dice Score of 0.757.

**Table 4:** Ablation study on AbdomenNet architecture. We report mean Dice scores and average symmetric surface distance per organ and the mean scores across organs

	Liver	Spleen	r.Kidney	l.Kidney	r.Adrenal	l.Adrenal	Pancreas	Gallbladder	Mean
*Dice coefficient*									
QuickNAT	0.948 ± 0.021	0.903 ± 0.043	**0.922** ± 0.018	**0.920** ± 0.023	0.503 ± 0.141	0.505 ± 0.185	0.733 ± 0.075	0.596 ± 0.206	0.754 ± 0.066
QuickNAT+Oct	**0.950** ± 0.020	0.907 ± 0.033	0.920 ± 0.015	0.916 ± 0.021	0.502 ± 0.161	0.492 ± 0.161	0.739 ± 0.097	0.671 ± 0.138	0.762 ± 0.059
AbdomenNet	**0.950** ± 0.019	**0.908** ± 0.034	0.921 ± 0.013	0.919 ± 0.023	**0.516** ± 0.155	**0.517** ± 0.136	**0.742** ± 0.086	**0.712** ± 0.146	**0.773** ± 0.054
*Average symmetric surface distance (mm)*									
QuickNAT	1.484 ± 1.005	1.373 ± 0.918	**0.623** ± 0.144	**0.652** ± 0.255	1.492 ± 0.514	1.916 ± 1.026	2.184 ± 0.748	2.873 ± 2.076	1.575 ± 0.500
QuickNAT+Oct	1.284 ± 0.835	1.220 ± 0.548	0.651 ± 0.131	0.815 ± 0.426	1.484 ± 0.673	1.825 ± 0.900	**2.006** ± 0.921	2.208 ± 1.203	1.437 ± 0.378
AbdomenNet	**1.206** ± 0.780	**1.191** ± 0.512	0.640 ± 0.129	0.801 ± 0.534	**1.355** ± 0.547	**1.667** ± 0.851	2.093 ± 0.938	**1.849** ± 1.206	**1.350** ± 0.350

Bold numbers indicate highest Dice scores and lowest ASSD scores

*Comparison with nn-UNet* We compare AbdomenNet to nn-UNet [29], which is a state-of-the-art segmentation network. We have trained a 2D nn-UNet model on the same training data as our joint AbdomenNet. As input to the nn-UNet pipeline, we used the already pre-processed data from our pre-processing pipeline. The 2D nn-UNet model achieved an average DSC of 0.75, which is slightly worse than the performance of AbdomenNet.

## Discussion

### Generalization

Our results have demonstrated that the generalization across datasets can be fairly poor. KORA and GNC share more similarities so that the generalization among those two datasets is better. Both datasets were acquired with 3T Siemens Skyra machines and also the scanning protocols were similar. In contrast, the generalization to UKB was clearly worse. A potential reason may, on the one hand, be related to the image acquisition, where a 1.5T Siemens Aera machine was used for UKB. On the other hand, also differences in the population may have an impact, as KORA and GNC are both studies in Germany. Also, the demographics presented in Table 1 are more similar for KORA and GNC.

Combining data from all datasets yielded the best results, even outperforming dataset-specific models. This is insofar surprising, as it is not clear that images from different datasets would rather cause confusion. The results indicate that the network has enough capacity to store dataset-specific information for multiple datasets, and therefore benefit from the increase in the size of the training set. Having a joint model that achieves high accuracy on each of the datasets is important as it supports studying diseases across datasets.

### Dixon contrasts

In the comparison among the four Dixon contrasts, the best results were obtained for OPP and water. Also by visual inspection, these two contrast provided the clearest depiction of the organs, see Fig. 1. Considering the results per organ, we note a considerable variation of the results between the different Dixon contrasts, which can also be observed in Fig. 1, e.g., it is difficult to detect the gallbladder in the Fat contrast. Our results have further shown that the combination of OPP and water as multi-channel input to the network can further improve results. An interesting observation is that the segmentation of kidneys improved significantly when using the combined OPP+W input, compared to single OPP or water inputs.

### Performance

AbdomenNet achieved high accuracy on liver, spleen, and both kidneys with Dice score above 0.9. In contrast, only a Dice score of around 0.5 was obtained for adrenal glands. The adrenal glands are complicated to segment because they are very small organs and only cover a few voxels. Figure 5 shows the small size of ground truth adrenal gland segmentation, and also shows that AbdomenNet was able to detect adrenal glands, but even small differences in the segmentations can lead to a reduced Dice score.

### Limitations

Our proposed pre-processing pipeline requires minimal human interaction by setting the cropping region once for each dataset. As we chose our cropping regions based on 3 large scale population studies, it already covers a variety of human body shapes, but it might need to be adapted for other datasets. In future work this could be automated, by extending the pipeline to localize relevant positions, e.g. as done by [37]. In this work we used manual annotations by a single rater as ground-truth labels. It would be interesting to gather annotations from multiple raters, to compare our network’s prediction performance with human inter-rater variability.

## Conclusions

We have presented a fully automated pipeline for the segmentation of abdominal organs across population studies. The proposed pre-processing pipeline standardizes images, which is necessary as several organs are only partially visible in the MRI scans from individual scanning stages. The standardization further reduces scanning artifacts in the images and therefore yields to a more homogeneous dataset, which facilitates learning. AbdomenNet achieved high accuracy for several organs, which supports future quantitative analyses of population studies. Our results have demonstrated the benefit of combining octave convolutions with squeeze and excite blocks in neural networks for organ segmentation. Finally, the joint segmentation model across datasets achieved higher accuracy than individual models per dataset.

## Data Availability

from UK Biobank: https://www.ukbiobank.ac.uk, KORA: https://www.helmholtz-munich.de/kora/ and GNC (NAKO): https://nako.de.

## Footnotes

1 https://github.com/ai-med/AbdomenNet.

## References

[1] Bamberg F, Kauczor H-U, Weckbach S, Schlett CL, Forsting M, Ladd SC, Greiser KH, Weber M-A, Schulz-Menger J, Niendorf T (2015). Whole-body MR imaging in the German national cohort: rationale, design, and technical background. Radiology.

[2] Streit F, Zillich L, Frank J, Kleineidam L, Wagner M, Baune BT, Klinger-König J, Grabe HJ, Pabst A, Riedel-Heller SG, Schmiedek F, Schmidt B, Erhardt A, Deckert J, Investigators N, Rietschel M, Berger K, Düsseldorf S, Leipzig S, Berlin-Süd S (2022). Lifetime and current depression in the German national cohort (NAKO). World J Biol Psychiatry.

[3] Littlejohns TJ, Holliday J, Gibson LM, Garratt S, Oesingmann N, Alfaro-Almagro F, Bell JD, Boultwood C, Collins R, Conroy MC (2020). The UK biobank imaging enhancement of 100,000 participants: rationale, data collection, management and future directions. Nat Commun.

[4] Hegenscheid K, Kühn JP, Völzke H, Biffar R, Hosten N, Puls R. Whole-body magnetic resonance imaging of healthy volunteers: pilot study results from the population-based ship study. In: RöFo-Fortschritte Auf dem Gebiet der Röntgenstrahlen und der Bildgebenden Verfahren, vol. 181; 2009. pp. 748–59. $$\copyright$$ Georg Thieme Verlag KG Stuttgart $$\cdot$$ New York. 10.1055/s-0028-1109510 19598074

[5] Bamberg F, Hetterich H, Rospleszcz S, Lorbeer R, Auweter SD, Schlett CL, Schafnitzel A, Bayerl C, Schindler A, Saam T (2017). Subclinical disease burden as assessed by whole-body MRI in subjects with prediabetes, subjects with diabetes, and normal control subjects from the general population: the KORA-MRI study. Diabetes.

[6] von Krüchten R, Lorbeer R, Müller-Peltzer K, Rospleszcz S, Storz C, Askani E, Kulka C, Schuppert C, Rathmann W, Peters A, Bamberg F, Schlett CL, Mujaj B (2022). Association between adipose tissue depots and dyslipidemia: the KORA-MRI population-based study. Nutrients.

[7] Fischl B, Salat DH, Busa E, Albert M, Dieterich M, Haselgrove C, Van Der Kouwe A, Killiany R, Kennedy D, Klaveness S (2002). Whole brain segmentation: automated labeling of neuroanatomical structures in the human brain. Neuron.

[8] Jenkinson M, Beckmann CF, Behrens TE, Woolrich MW, Smith SM (2012). Fsl. Neuroimage.

[9] Wachinger C, Rieckmann A, Pölsterl S (2021). Detect and correct bias in multi-site neuroimaging datasets. Med Image Anal.

[10] Ronneberger O, Fischer P, Brox T. U-net: convolutional networks for biomedical image segmentation. In: International conference on medical image computing and computer-assisted intervention. Springer; 2015. pp. 234–41.

[11] Roy AG, Conjeti S, Navab N, Wachinger C (2019). Quicknat: a fully convolutional network for quick and accurate segmentation of neuroanatomy. NeuroImage.

[12] Chen Y, Fan H, Xu B, Yan Z, Kalantidis Y, Rohrbach M, Yan S, Feng J. Drop an octave: reducing spatial redundancy in convolutional neural networks with octave convolution. In: Proceedings of the IEEE/CVF international conference on computer vision; 2019. pp. 3435–44.

[13] Hu J, Shen L, Sun G. Squeeze-and-excitation networks. In: CVPR; 2018. pp. 7132–41.

[14] Roy AG, Navab N, Wachinger C (2019). Recalibrating fully convolutional networks with spatial and channel ‘squeeze and excitation’ blocks. IEEE TMI.

[15] Izmailov P, Podoprikhin D, Garipov T, Vetrov D, Wilson AG. Averaging weights leads to wider optima and better generalization; 2018. arXiv:1803.05407.

[16] Sezgin M, Sankur B (2004). Survey over image thresholding techniques and quantitative performance evaluation. J Electron Imaging.

[17] Pohle R, Toennies KD. Segmentation of medical images using adaptive region growing. In: Medical imaging 2001: image processing, vol. 4322. SPIE; 2001. pp. 1337–46.

[18] Park H, Bland PH, Meyer CR (2003). Construction of an abdominal probabilistic atlas and its application in segmentation. IEEE Trans Med Imaging.

[19] Roth HR, Oda H, Hayashi Y, Oda M, Shimizu N, Fujiwara M, Misawa K, Mori K. Hierarchical 3d fully convolutional networks for multi-organ segmentation; 2017. arXiv:1704.06382. 10.1016/j.compmedimag.2018.03.001 29573583

[20] Wang Y, Zhou Y, Shen W, Park S, Fishman EK, Yuille AL (2019). Abdominal multi-organ segmentation with organ-attention networks and statistical fusion. Med Image Anal.

[21] Zhou Y, Li Z, Bai S, Wang C, Chen X, Han M, Fishman E, Yuille AL. Prior-aware neural network for partially-supervised multi-organ segmentation. In: Proceedings of the IEEE/CVF international conference on computer vision; 2019. pp. 10672–81.

[22] Gibson E, Giganti F, Hu Y, Bonmati E, Bandula S, Gurusamy K, Davidson B, Pereira SP, Clarkson MJ, Barratt DC (2018). Automatic multi-organ segmentation on abdominal CT with dense v-networks. IEEE Trans Med Imaging.

[23] Chen Y, Ruan D, Xiao J, Wang L, Sun B, Saouaf R, Yang W, Li D, Fan Z (2020). Fully automated multi-organ segmentation in abdominal magnetic resonance imaging with deep neural networks. Med Phys.

[24] Bobo MF, Bao S, Huo Y, Yao Y, Virostko J, Plassard AJ, Lyu I, Assad A, Abramson RG, Hilmes MA. Fully convolutional neural networks improve abdominal organ segmentation. In: Medical imaging 2018: image processing, vol. 10574. International Society for Optics and Photonics; 2018. p. 105742. 10.1117/12.2293751 PMC5992909 29887665

[25] Rickmann A-M, Roy AG, Sarasua I, Wachinger C (2020). Recalibrating 3d convnets with project & excite. IEEE Trans Med Imaging.

[26] Senapati J, Roy AG, Pölsterl S, Gutmann D, Gatidis S, Schlett C, Peters A, Bamberg F, Wachinger C. Bayesian neural networks for uncertainty estimation of imaging biomarkers. In: International workshop on machine learning in medical imaging. Springer; 2020. pp. 270–80.

[27] Jimenez-del-Toro O, Müller H, Krenn M, Gruenberg K, Taha AA, Winterstein M, Eggel I, Foncubierta-Rodríguez A, Goksel O, Jakab A (2016). Cloud-based evaluation of anatomical structure segmentation and landmark detection algorithms: visceral anatomy benchmarks. IEEE Trans Med Imaging.

[28] Kavur AE, Gezer NS, Barış M, Aslan S, Conze P-H, Groza V, Pham DD, Chatterjee S, Ernst P, Özkan S (2020). Chaos challenge-combined (CT-MR) healthy abdominal organ segmentation. Med Image Anal.

[29] Isensee F, Jaeger PF, Kohl SA, Petersen J, Maier-Hein KH (2020). nnU-net: a self-configuring method for deep learning-based biomedical image segmentation. Nat Methods.

[30] Tustison NJ, Avants BB, Cook PA, Zheng Y, Egan A, Yushkevich PA, Gee JC (2010). N4itk: improved n3 bias correction. IEEE Trans Med Imaging.

[31] Basty N, Liu Y, Cule M, Thomas EL, Bell JD, Whitcher B. Image processing and quality control for abdominal magnetic resonance imaging in the UK biobank; 2020. arXiv:2007.01251.

[32] Noh H, Hong S, Han B. Learning deconvolution network for semantic segmentation. In: ICCV; 2015. pp. 1520–8.

[33] Huang G, Liu Z, Van Der Maaten L, Weinberger KQ. Densely connected convolutional networks. In: Proceedings of the IEEE conference on computer vision and pattern recognition; 2017. pp. 4700–8.

[34] He K, Zhang X, Ren S, Sun J. Delving deep into rectifiers: surpassing human-level performance on imagenet classification. In: Proceedings of the IEEE international conference on computer vision; 2015. pp. 1026–34.

[35] Wu Y, He K. Group normalization. In: Proceedings of the European conference on computer vision (ECCV); 2018. pp. 3–19.

[36] Ulyanov D, Vedaldi A, Lempitsky V. Improved texture networks: maximizing quality and diversity in feed-forward stylization and texture synthesis. In: CVPR; 2017. pp. 6924–32.

[37] Estrada S, Lu R, Conjeti S, Orozco-Ruiz X, Panos-Willuhn J, Breteler MM, Reuter M (2020). Fatsegnet: a fully automated deep learning pipeline for adipose tissue segmentation on abdominal dixon MRI. Magn Reson Med.

